# Cardiovascular magnetic resonance can detect occult anthracycline cardiotoxicity in adolescents and young adults cancer survivors with normal ejection fraction

**DOI:** 10.1186/1532-429X-18-S1-Q42

**Published:** 2016-01-27

**Authors:** Miguel S Vieira, Isma Rafiq, Alberto C Figueroa, Sujeev K Mathur, Tarique Hussain

**Affiliations:** 1 grid.13097.3c0000000123226764Division of Imaging Sciences & Biomedical Engineering, King's College London British Heart Foundation Centre, London, United Kingdom; 2 grid.214458.e0000000086837370Departments of Surgery and Biomedical Engineering, University of Michigan, Ann Arbor, MI USA; 3 Pediatric Cardiology, University of Texas, Dallas, TX USA; 4 Pediatric Cardiology, Evelina London Children's Hospital/Guy's and St Thomas' NHS Foundation Trust, London, United Kingdom

## Background

Risk stratification and early monitoring of anthracycline-induced cardiotoxicity is crucial. It has been shown that early histopathological changes occur well before myocardial functional abnormalities can be detected. Additionally a long period of latency with clinical recovery of systolic function before the development of overt heart failure has been described, further emphasizing the importance of early recognition of cardiac injury and defining routine follow-up.

## Methods

Long-term childhood cancer survivors were prospectively recruited after written informed consent. Study protocol was approved by the institutional review board. Subjects underwent a cardiovascular magnetic resonance (CMR) study on a 1.5 Tesla scanner (Philips, Best, Netherlands) with routine cine acquisition in long and short axis planes, tissue characterization with native T1 mapping and late gadolinium enhancement (LGE), selected 2D phase contrast flow imaging and 3D whole heart anatomy. Ventricular volumes, mass, and ejection fraction (EF) were obtained via standard planimetry techniques using a semiautomated commercial software (CMRtools, Imperial College, London, United Kingdom). Myocardial and left atrial (LA) strain parameters were derived from balanced SSFP cine images using dedicated software (TomTec Imaging Systems, 2D CPA MR, Cardiac Performance Analysis, Version 1.1.2.36, Unterschleissheim, Germany). LA reservoir, conduit and contractile functions were then quantified by both fractional volume changes and CMR feature tracking derived strain and strain rate. Transit times for pulse wave velocity (PWV) computations were determined using the foot-to-foot methodology.

## Results

**Figure 1:**
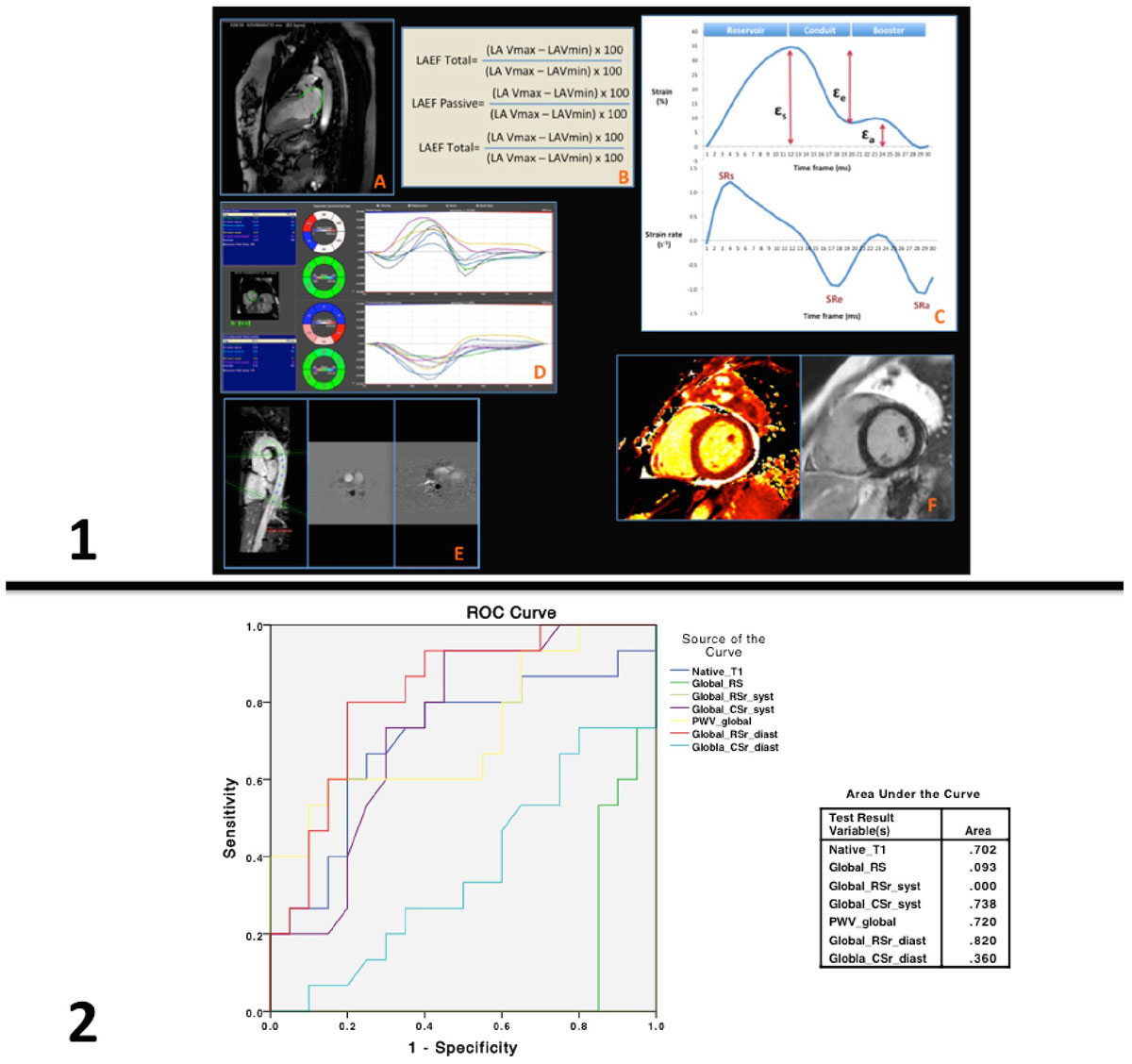
**Panel 1 (A to F) depicts the PWV, T1 mapping/myocardial fibrosis analysis, LA and LV indices assessed**. ROC curve (Panel 2) comparing the sensitivity and specificity of different imaging biomarkers analyzed and chemotherapy treatment.

Twenty patients (mean age 18 ± 2 years) were enrolled. Twenty age-matched healthy controls (p = 0.109) were also recruited for comparison. Patients were studied after a mean anthracycline cumulative dose of 207 mg/m^2^, 83 months on average post-exposure. There was no significant difference between both groups in terms of volumetric analysis (table 1), with both presenting no areas of myocardial LGE. Left ventricular deformation parameters (but not LA functional indices) such as global radial strain (GRs) and strain rate (GRSr), global circumferential strain (GCs) and strain rate (GCSr), native T1 and PWV were significantly different among the two groups on both bivariate and multivariate regression analysis (p <0.001 to 0.035). GRSr (early diastole), GCSr (systole), PWV and native T1 outperform other imaging biomarkers (figure 1) in the assessment of subtle myocardial and vascular changes post-chemotherapy.

**Table 1:** Baseline characteristics

CHARACTERISTICS	PATIENTS	CONTROLS	p
Age, years	18 ± 2 [12-21]	19 ± 3 [12-24]	0.109
Gender, n	11 males; 9 females	12 males; 8 females	0.749
LV ejection fraction [LVEF (%)]	59 ± 3.5	61 ± 4.2	0.057
LV end-diastolic volume indexed [LVEDVi (mL/m2)]	85 ± 16.5	85 ± 14.1	0.975
LV end-systolic volume [LVESVi (mL/m2)]	35 ± 9.5	35 ± 9.1	0.946
Global circumferential strain (GCs)	-14.0 ± 3.6	-16.4 ± 3.5	0.041
Global radial strain (GRs)	17.2 ± 6.4	30.7 ± 6.9	<0.001
Global circumferential strain rate [GCSr (syst)]	-1.25 ± 0.26	-1.58 ± 0.41	0.005
Global radial strain rate peak systole [GCRSr (syst)]	0.86 ± 0.21	2.44 ± 0.5	<0.001
Global circumferential strain rate early diastole [GCSr (diast)]	1.49 ± 0.41	1.85 ± 0.52	0.022
Global radial strain rate early diastole [GRSr (diast)]	-1.61 ± 0.38	-2.21 ± 0.61	0.001
Native T1 (mid septum, ms)	1004 ± 70.6	966 ± 30.6	0.035
LA ejection fraction total (%)	66.9 ± 8.3	67.9 ± 7.8	0.692
LA conduit function (%)	41.2 ± 15.1	41.5 ± 10.6	0.928
LA booster pump function (%)	42.3 ± 9.1	44.5 ± 10.8	0.682
LA total strain (εs)	30.6 ± 8.6	30.7 ± 7.4	0.967
LA passive strain (εe)	24.1 ± 6.4	23.3 ± 5.2	0.638
LA active strain (εa)	6.5 ± 3.5	7.5 ± 3.6	0.400
LA peak positive strain rate (SRs)	1.2 ± 0.3	1.3 ± 0.4	0.430
LA peak early negative strain rate (SRe)	-1.7 ± 0.4	1.7 ± 0.4	0.941
LA peak late negative strain rate (SRa)	-0.9 ± 0.3	-1.0 ± 0.3	0.148
Pulse wave velocity [PWV (m/s)]	4.605 ± 0.51	3.742 ± 0.23	0.019

## Conclusions

CMR can detect subtle myocardial and vascular abnormalities in young cancer survivors with normal EF exposed to anthracycline chemotherapy in the months following exposure. CMR may provide the short term markers of future cardiac morbidity necessary to design safer treatment regimens for younger cancer survivors.

